# Dr. Herbert Mickle

**Published:** 1903-08

**Authors:** 


Dr. Herbert Mickle, of Cleveland, formerly of Buffalo, died at Asheville, N. C., July 22, 1903, aged 43 years. He was born at Guelph, Ont., and was educated at Upper Canada College and Trinity Medical School, Toronto, and Saint Bartholomew's Hospital Medical School, London, Eng. He established himself in Buffalo and was at once appointed surgeon to the Emergency Hospital and demonstrator of anatomy in the Niagara University. Later, he became professor of surgery in Niagara University and attending surgeon at the Sisters' Hospital, Saint Francis's Hospital, Emergency Hospital and the Church Home. He lived at No. 526 Delaware Avenue. Two years ago he became afflicted with lung trouble and recently gave up professional practice and accepted the office of medical examiner for the New York Life Insurance Company at Cleveland.

Dr. Mickle is survived by a widow, who was with him when he died.

